# Repurposing Verapamil to Enhance Killing of T-ALL Cells by the mTOR Inhibitor Everolimus

**DOI:** 10.3390/antiox12030625

**Published:** 2023-03-03

**Authors:** Micol Silic-Benussi, Evgeniya Sharova, Alberto Corradin, Loredana Urso, Vittoria Raimondi, Ilaria Cavallari, Barbara Buldini, Samuela Francescato, Sonia A. Minuzzo, Donna M. D’Agostino, Vincenzo Ciminale

**Affiliations:** 1 Veneto Institute of Oncology IOV—IRCCS, 35128 Padova, Italy; 2 Department of Surgery, Oncology and Gastroenterology, University of Padova, 35128 Padova, Italy; 3 Pediatric Hemato Oncology, Maternal and Child Health Department, University of Padova, 35128 Padova, Italy; 4 Department of Biomedical Sciences, University of Padova, 35131 Padova, Italy

**Keywords:** ROS, leukemia, cell death, pentose phosphate pathway

## Abstract

New therapies are needed for patients with T-cell lymphoblastic leukemia (T-ALL) who do not respond to standard chemotherapy. Our previous studies showed that the mTORC1 inhibitor everolimus increases reactive oxygen species (ROS) levels, decreases the levels of NADPH and glucose-6-phosphate dehydrogenase (G6PD), the rate-limiting enzyme of the pentose phosphate pathway (PPP), and induces apoptosis in T-ALL cells. Studies in T-ALL-xenografted NOD/SCID mice demonstrated that everolimus improved their response to the glucocorticoid (GC) dexamethasone. Here we show that verapamil, a calcium antagonist used in the treatment of supraventricular tachyarrhythmias, enhanced the effects of everolimus on ROS and cell death in T-ALL cell lines. The death-enhancing effect was synergistic and was confirmed in assays on a panel of therapy-resistant patient-derived xenografts (PDX) and primary samples from T-ALL patients. The verapamil-everolimus combination produced a dramatic reduction in the levels of G6PD and induction of p38 MAPK phosphorylation. Studies of NOD/SCID mice inoculated with refractory T-ALL PDX cells demonstrated that the addition of verapamil to everolimus plus dexamethasone significantly reduced tumor growth in vivo. Taken together, our results provide a rationale for repurposing verapamil in association with mTORC inhibitors and GC to treat refractory T-ALL.

## 1. Introduction

Approximately 25% of T-cell acute lymphoblastic leukemia (T-ALL) patients are resistant/refractory to current therapy (R/R-T-ALL) [1]. Glucocorticoids (GC) are the cornerstone of T-ALL therapy, and response to GC is an important predictor for prognostic stratification of the patients. Overcoming resistance to GC is thus a deciding factor for the successful treatment of T-ALL [2]. 

T-ALL cells are characterized by a rewiring of the homeostatic set point of reactive oxygen species (ROS) [3,4], which increases the vulnerability of these cells to ROS-inducing stimuli [5,6]. We previously showed that pharmacological inhibition of mTORC1 using everolimus or genetic silencing of components of this pathway results in a sustained elevation of ROS levels and primes T-ALL cells to apoptosis [7]. Interestingly, mTOR inhibition resulted in a decrease in the levels of glucose-6-phosphate dehydrogenase (G6PD), the rate-limiting enzyme of the pentose phosphate pathway (PPP), which is a principal source of cytosolic NADPH, an electron donor needed to maintain ROS scavengers in their reduced/active state. Furthermore, the combination of everolimus with the GC dexamethasone effectively killed GC-resistant T-ALL cells both in vitro and in a preclinical model that employed patient-derived xenografts (PDX) of T-ALL cells in NOD/SCID mice.

Clinical trials tested an mTOR inhibitor (everolimus or tensirolimus) in association with chemotherapy in R/R T-ALL patients [8]. Unfortunately, the results of these trials demonstrated limited efficacy of the studied drug combinations. A seminal study reported that a wide range of drug-resistant cancer cell types express the multidrug efflux pump P-glycoprotein (P-gp) [9]. P-gp is a member of the ATP-binding cassette (ABC) transporter superfamily and is coded by the ABCB1 (MDR1) gene. We reasoned that increased drug efflux through multidrug resistance (MDR) pumps might significantly blunt the efficacy of mTOR inhibitors in patients. To test this hypothesis, we treated R/R T-ALL cells with everolimus, dexamethasone, and verapamil, a calcium channel antagonist that is known to also inhibit several MDR pumps of the ABC superfamily [10]. 

## 2. Material and Methods

### 2.1. T-ALL Cell Lines and Primary Samples

Primary T-ALL cells and the T-ALL-derived cell lines Jurkat, TALL-1, CCRF-CEM, and DND41 were cultured in RPMI 1640 medium (Euroclone) supplemented with 10% fetal calf serum (FCS), 2 mM L-glutamine, 100 units/mL penicillin, and 20 units/mL streptomycin. PDX stabilized from primary pediatric T-ALL samples were propagated in NOD/SCID mice. The xenograft cells were recovered from the spleens of mice that developed leukemia and used in in vitro assays as previously described [7,11,12]. Primary samples from T-ALL patients were obtained in compliance with the Code of Ethics of the World Medical Association Declaration of Helsinki in the context of the AIEOP BFM ALL 2009 protocol. Informed consent was obtained from patients or their legal representatives.

Cells (1 × 10^6^/mL) were treated for 24 h with either ethanol (0.1% final concentration, control), everolimus (Selleck Chemicals Houston, TX, USA), verapamil (Merck KGaA, Darmstadt, Germany), or everolimus plus verapamil at the concentrations indicated in the figure legends, and then analyzed as described below. Unless otherwise indicated, all cell treatments were performed in a 37 °C, 5% CO_2_ incubator.

### 2.2. RNA-seq and Bioinformatic Analysis

The present study employed RNA-seq data obtained for poly-A-enriched RNA isolated from Jurkat, TALL-1, and CEM cells after 24 h of treatment with 10 μM everolimus or drug vehicles [7]. The Wald statistical test was performed using DeSeq2 and APEGLM shrinkage [13] to identify transcripts with altered expression upon everolimus treatment; *p*-values were adjusted for multiple tests [14]. Genes showing a fold change >2 and padj < 0.05 were classified as differentially expressed. Expression data for genes in the ABC transporter family [15] were used to construct volcano plots.

### 2.3. Cell Death Assay

Cell death was measured by staining 2 × 10^5^ cells with propidium iodide (PI, 2 µg/mL, Sigma-Aldrich) for 10 min at room temperature. PI staining was detected using a FACSCelesta flow cytometer (BD Biosciences, San Jose, CA, USA). Data from 10,000 ungated events were elaborated using the Kaluza software (Beckman-Coulter Franklin Lakes, NJ, USA), and used to calculate Specific cell death (SCD) as follows:

SCD = [(% of dead cells in drug-treated sample − % of dead cells in mock-treated control)/(100 − % of dead cells in mock-treated control)] × 100.(1)

### 2.4. Synergy Study

The possible synergistic effect of the everolimus plus verapamil combination was evaluated using the Bliss model and the Combenefit platform [16]. T-ALL cell lines were treated with increasing concentrations of everolimus (0–15 µM) and verapamil (0–30 ng/mL), as well as in combination; after 24 h of treatment, cell death was measured by PI staining. Data obtained from the cell death assay were used to construct a matrix (6 × 6), which was elaborated with a Combenefit software version 2.021. Isobologram analysis was also used to confirm the synergistic interaction of everolimus and verapamil [17].

### 2.5. Mass Spectrometry and Quantification of Intracellular Levels of Everolimus

Cells were treated for 24 h with 10 µM everolimus alone or in combination with the MDR inhibitors verapamil (20 ng/mL) or MK 571 (10 µM). Cells were then collected, washed with PBS, and lysed in cell disruption buffer [(25 mM Tris-HCl, 150 mM NaCl, 1 mM EDTA, 1% NP-40, 5% glycerol, pH 7.4) containing inhibitors of phosphatases and proteases (PhosphoSTOP and cOmplete, Roche, Basel, Switzerland)]. A 30 µL sample of lysate was mixed with 70 µL of methanol, sonicated for 20 min, and then centrifuged for 10 min at 10,000 rpm to precipitate proteins. The supernatant was collected and subjected to multiple reaction monitoring (MRM) and mass spectrometry (MS) with a 4000 QTrap system. The analysis was performed by Cytosens Srls., Milan.

### 2.6. Hoechst 33342 Exclusion Assay

The MDR pump activity of T-ALL cells was measured using a method based on the retention/efflux of the fluorescent DNA-binding dye Hoechst 33342 [18] as follows: cells were treated with the MDR inhibitors verapamil (20 ng/mL) or MK 571 (10 µM) for 30 min. The cells were then labeled with Hoechst 33342 (8 µM) at room temperature for 30 min, centrifuged, re-suspended in PBS, and immediately analyzed using a FACSCelesta flow cytometer. Samples were analyzed using a 405-nm excitation filter and emissions at 450 and 670 nm (blue and red signals, respectively). Scatter plots with blue and red emission signals plotted on the y and x axes were generated with the Kaluza software, and an arbitrary gate was applied to all plots to compare the percentages of ‘Hoechst-dim’ cells (i.e., cells that effluxed the dye) in the different samples.

### 2.7. Analysis of ROS Production in Living Cells

Cells were incubated with MitoSOX Red (2.6 µM, Thermo Fisher Scientific, Waltham, MA, USA) and CellRox Deep Red (5 µM, Thermo Fisher Scientific) for 30 min. Fluorescent signals were detected using a FACSCelesta flow cytometer and Diva software (BD Biosciences) to obtain mean fluorescence intensity (MFI) values in the live-cell gate. Changes in ROS were expressed as Fx/F0 ratios, where Fx corresponds to the MFI of each sample and F0 is the MFI of the “no drug” control.

### 2.8. Lipid Peroxidation Assay

Cells were incubated with the Image-iT lipid peroxidation sensor (2 µM, Thermo Fisher Scientific) for 30 min and then analyzed with a FACSCelesta flow cytometer to detect signals from the oxidized probe (green fluorescence emission peak at 510 nm) and total probe (red fluorescence emission peak at 590 nm). Lipid peroxidation was calculated as the ratio between the oxidized- and total-probe signals. 

### 2.9. Immunoblotting and Protein Oxidation Status

Cells were collected, washed with PBS, and lysed in cell disruption buffer. The protein concentration was determined with the Bradford protein assay (Quick Start™, Bio-Rad, Hercules, CA, USA). A 30 µL sample of protein was separated by SDS/PAGE in 4–20% mini-PROTEAN TGX precast gradient gels (BioRad, Hercules, CA, USA) and then transferred to nitrocellulose membranes (GE Healthcare, Chicago, IL, USA). The membranes were saturated with 3% bovine serum albumin (BSA) in TBS (50 mM Tris-HCl, pH 7.5, 150 mM NaCl)-0.01% Tween-20 and then incubated overnight in the Purity Western Blot Detection System reagent (Vilber Lourmat, Marne-la-Vallée, France) containing the primary antibodies (1:1000 dilution) and the appropriate secondary anti-rabbit/anti-mouse HRP-conjugated antibodies. Primary antibodies included mouse anti-phospho-p38 MAPK Thr180/Tyr182 (9216, Cell Signaling Technology, Danvers, MA, USA), rabbit anti-p38 MAPK (9212, Cell Signaling Technology), and mouse anti-G6PD (sc-373886, Santa Cruz Biotechnology, Dallas, TX, USA). Membranes were washed twice, and chemiluminescent signals were detected using the Lite Ablot Turbo (EuroClone, Pero (MI), Italy) and a Cambridge UVITEC imaging system. The same membranes were then probed with rabbit anti-GAPDH antibodies (GTX100118, GeneTex, Irvine, CA, USA, 1:1000 dilution, 1 h), followed by HRP-conjugated anti-rabbit antibodies (1:5000, Thermo Fisher Scientific), and developed as described above. The cell oxidation status was analyzed with the OxyBlot Protein Oxidation Detection Kit (S7150, Merck), as described in the legend to Figure S2.

### 2.10. In Vivo Experiments

All procedures involving animals and their care were authorized by the ethics committees of the University of Padova and the Italian Ministry of Health (authorization no. 876/2018-PR) in compliance with the ARRIVE guidelines and European Union directives (86/609/EEC and 2010/63/EU). In vivo experiments were performed on NOD/SCID mice housed in a specific pathogen-free (SPF) facility under conditions that ensured the animals’ well-being, as described in ref. [7]. Mice were anesthetized with isoflurane prior to blood sampling and injections. Manipulations were performed by selecting mice in a random order. 

Mice (6–8-week-old females, weighing approximately 20 g) were inoculated with 1 × 10^6^ T-ALL PDX19 cells by injection into the tail vein (day T = 0). One week later, mice were randomly divided into experimental groups, tagged for identification, and injected intraperitoneally (IP) with either 50% polyethylene glycol (PEG)/50% H_2_O (vehicle control), dexamethasone (15 mg/kg), everolimus (4 mg/kg), or dexamethasone (15 mg/kg) + everolimus (4 mg/kg); treatments were repeated every second day. Verapamil (20 mg/kg) was added to the treatment when the blasts in the blood were >10%.

Blood samples were obtained from the submandibular plexus. The experimental endpoint (circulating tumor cells representing >50% of total PBMC) was chosen to minimize animal suffering. After being anesthetized with isoflurane, mice were euthanized with CO_2_.

### 2.11. Statistical Analysis and Graphics

Graphs were constructed using SigmaPlot. Figures were generated using CorelDraw, Power Point, and elements from BioRender.com. The statistical tests employed to assess significance are indicated in the figure legends.

## 3. Results

### 3.1. Expression of ABC Transporters in T-ALL Cells

**Figure 1:**
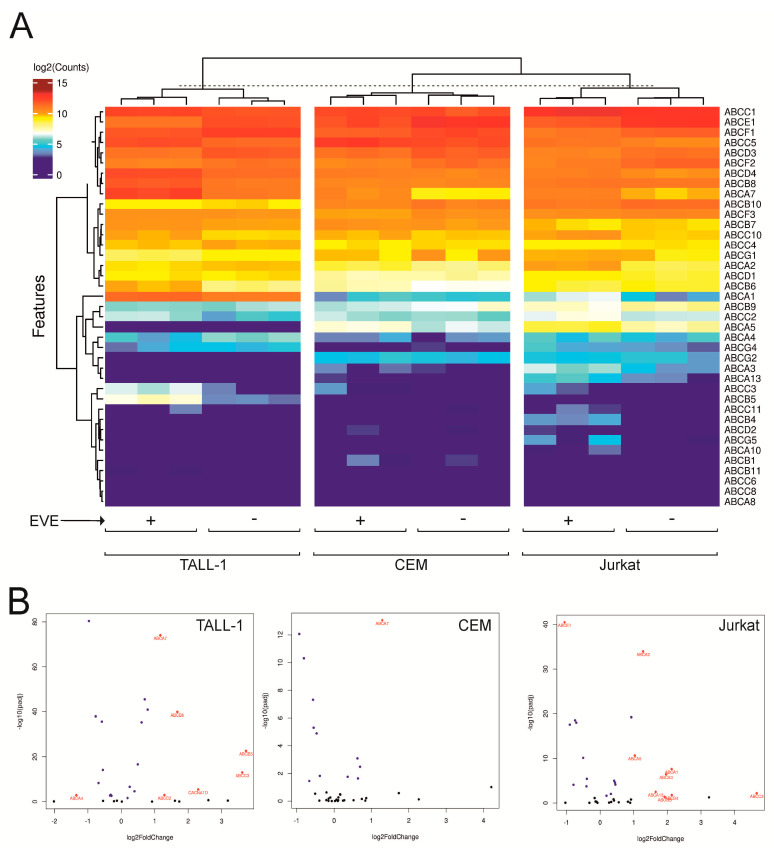
RNA-seq analysis of T−ALL cells. (**A**) A heat map of supervised cluster analysis showing normalized reads for ABC family genes [15] in the cell lines Jurkat, TALL−1, and CEM in logarithmic scale. (**B**) Volcano plots show genes of the custom ABC family gene set. Blue color indicates statistical significance of the feature; red color indicates differential expression (padj < 0.05 and FC > 2), referring to comparisons of “treated versus untreated” samples.

As a starting point of our study regarding the role of MDR pumps in the response of T-ALL cells to everolimus, we investigated the expression pattern of ABC transporters in T-ALL cell lines using an RNA-seq. We generated a custom gene set including all the members of the ABC family [15]. Supervised cluster analysis showed a similar overall pattern of expression of ABC family genes among the lines (Figure 1A); interestingly, ABCC1, the top-scoring gene in terms of expression, is a known target of verapamil, a well-known inhibitor of MDR activity [10]. Furthermore, treatment with everolimus resulted in a significant upregulation of ABCA1, ABCA2, ABCA3, ABCA5, ABCA13, ABCB4, ABCC3, and ABCG5 (and ABCE1 downregulation) in Jurkat cells; ABCA7, ABCB5, ABCB6, and ABCC2, ABCC3 were upregulated, and ABCA4 was downregulated in TALL-1 cells; only ABCA7 was slightly upregulated in CEM cells (Figure 1B).

### 3.2. Verapamil Enhances the Killing Effect of Everolimus in T-ALL Cells

**Figure 2:**
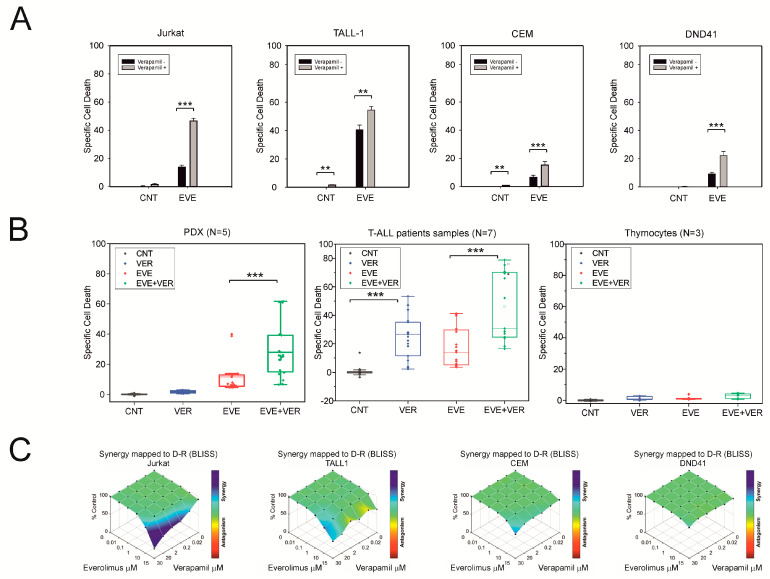
Verapamil enhances cell death induced by everolimus. (**A**) Plots of specific cell death assessed by PI staining in T-ALL cell lines treated for 24 h with verapamil (VER, 20 ng/mL), everolimus (EVE, 10 µM), or with both drugs. (**B**) Plots of specific cell death induced by a 24-h treatment with VER (20 ng/mL), EVE (10 µM), or both drugs in five T-ALL PDX, primary tumor cells from T-ALL patients (*N* = 7), and three preparations of normal thymocytes; mean values and standard error bars are shown. The indicated pairwise comparisons were statistically significant with the Mann-Whitney test; *** indicate *p* values < 0.001, ** < 0.01. (**C**) Combinatory effects of EVE and VER on T-ALL cells. Shown are Combenefit-generated surface maps for drug combinations in T-ALL cell lines. The axes indicate concentrations of each drug.

Next, we tested the role of MDR pumps in the response of T-ALL cells to everolimus by adding verapamil. Results showed that verapamil enhanced the cell death effects of everolimus in four T-ALL cell lines (Jurkat, TALL−1, CEM, and DND41; Figure 2A), five PDX (Figure 2B, left), and in leukemia cells from seven patients (Figure 2B, middle). Notably, these drug combinations were not toxic for normal, freshly isolated thymocytes (Figure 2B, right). Interestingly, verapamil alone exhibited a powerful cytotoxic effect on primary T-ALL cells (Figure 2B) and a modest but significant effect on TALL−1 and CEM cells (Figure 1A). The functional interaction of everolimus and verapamil in T-ALL cells was analyzed with the Bliss model and Combenefit software [16]. The surface output for drug combination results showed that the interaction of these drugs was highly synergistic in Jurkat and TALL-1 cells, but less so in the CEM and DND41 lines (Figure 2C). 

The synergistic interaction of these compounds was confirmed by the Chou-Thalalay isobologram analysis (Figure S1).

### 3.3. Verapamil does Not Significantly Change the Intracellular Concentration of Everolimus

**Figure 3:**
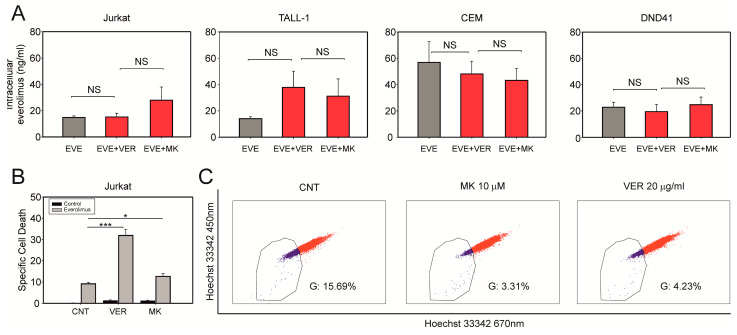
In vitro effects of MDR inhibitors. (**A**) Quantification of everolimus in the four T-ALL cell lines treated with everolimus (EVE, 10 µM) alone, or in combination with MK 571 (10 µM) or verapamil (VER, 20 ng/mL). Everolimus was quantified by MRM-MS and the 4000 QTrap system. (**B**) Specific cell death after a 24-h treatment with verapamil (20 ng/mL) or MK 571 (10 µM) combined with EVE (10 µM); mean values and standard error bars are shown. The indicated pairwise comparisons were statistically significant with the Mann-Whitney test; *** indicate *p* values < 0.001, * < 0.05, NS = not significant. (**C**) Inhibition of Hoechst 33342 efflux by verapamil and MK 571. Dot blots show blue and red fluorescence (emission at 450 and 670 nm, respectively) of Jurkat cells stained with the MDR substrate Hoechst 33342 (8 µM) after a 30-min treatment with MDR inhibitors. The three graphs report percentages of cells with low fluorescence levels (i.e., those that have excluded the dye) measured in the same arbitrary gate.

Next, we tested the effects of verapamil on the intracellular concentration of everolimus. Mass spectrometry analysis of lysates prepared from the four T-ALL cell lines treated with everolimus did not reveal significant changes in the intracellular concentration of everolimus in response to verapamil or MK 571, another established MDR inhibitor (Figure 3A). A control flow cytometry experiment confirmed that both verapamil and MK 571 inhibited MDR activity, as they produced a decrease in the percentage of ‘Hoechst 33342-dim’ cells (Figure 2C). It is worth noting that although both verapamil and MK 571 enhanced the death-inducing effect of everolimus, verapamil appeared to be much more powerful (Figure 3B). Taken together, these findings suggest that everolimus is not a substrate of the MDR in the T-ALL cell lines and that the enhancement of everolimus-induced cell death is not likely to reflect MDR inhibition.

### 3.4. Verapamil Enhances the Effects of Everolimus on Redox Homeostasis

**Figure 4:**
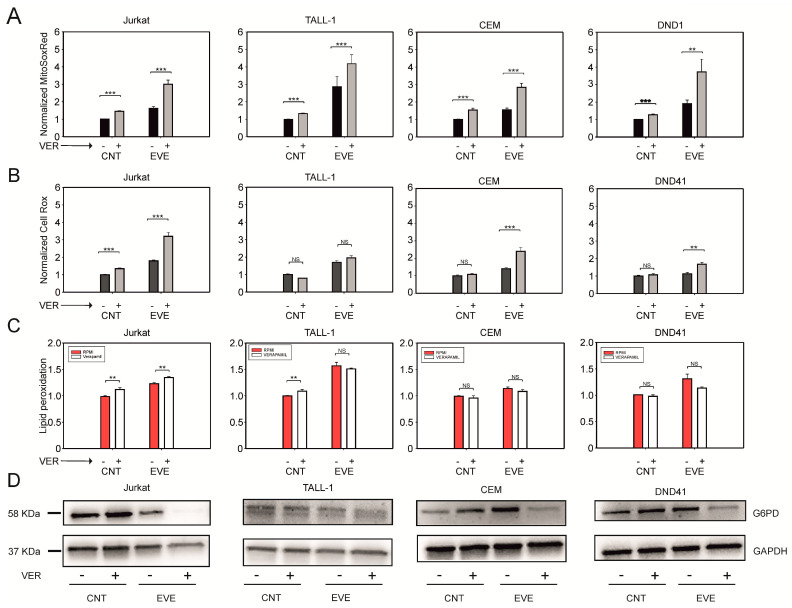
Verapamil enhances the perturbation of redox homeostasis induced by everolimus. (**A**) Plots of mean fluorescence intensity (MFI) values for the MitoSOX rRed fluorescent probe measured after 24 h of everolimus (EVE)/verapamil (VER) treatment and scaled against MFI values measured in the control cells. (**B**) Plots of MFI values for the CellRox Deep Red fluorescent probe measured after 24 h of everolimus/verapamil treatment and scaled against MFI values measured in the control cells. (**C**) Lipid peroxidation in T-ALL cells treated for 24 h with everolimus and verapamil. Shown are mean lipid peroxidation values (measured with the Image-iT Lipid peroxidation sensor). The mean values and standard error bars of three independent experiments with three replicates each obtained in Jurkat, TALL−1, CEM and DND41 cells are shown. The indicated pairwise comparisons were statistically significant with the Mann-Whitney test; *** indicate *p* values < 0.001, ** < 0.01, NS = not significant. (**D**) G6PD expression in T-ALL cells treated with EVE (10 µM) and/or VER (20 ng/mL). Shown are immunoblots from representative experiments.

Our previous studies showed that everolimus treatment of T-ALL cells induces an increase in their ROS levels and that the cytotoxic effects of everolimus on T-ALL cells are ROS-dependent [7]. Therefore, we investigated the effects of verapamil alone, and in combination with everolimus, on ROS levels. We treated Jurkat, TALL−1, CEM, and DND41 cells with the different drug combinations for 24 h and measured ROS with MitoSOX Red (Figure 4A), which is specific for the mitochondrial compartment, and CellROX Deep Red (Figure 4B), which provides a ‘whole cell’ measurement. Results showed that verapamil alone increased mitochondrial ROS levels and potentiated the effect of everolimus in all four cell lines (Figure 4A). Analogous measurements of the ‘whole cell’ ROS revealed a significant effect of verapamil in Jurkat cells and a potentiation of the effect of everolimus in Jurkat, CEM, and DND41 cells (Figure 4B). The impact of verapamil on cellular redox status was confirmed with an assay that measured intracellular H_2_O_2_ and with OxyBlots to detect carbonyl groups, a hallmark of protein oxidation status (Figure S2). Verapamil also induced a modest but significant increase in the levels of lipid peroxidation in Jurkat and TALL−1 cells but not in CEM or DND41 cells (Figure 4C). 

Our previous studies indicated that everolimus induces a decrease in levels of the PPP enzyme G6PD [7]. Therefore, we tested the effects of verapamil alone or in combination with everolimus on G6PD levels. To better visualize the functional interaction of these two compounds, we used everolimus at a concentration of 10 µM, which, as a single agent, produces a partial decrease in G6PD levels [7]. Results of immunoblot analysis showed that the combination of everolimus and verapamil produced a dramatic reduction in G6PD levels (Figure 4D and Figure S3).

### 3.5. Verapamil and Everolimus Induce Phosphorylation of p38 MAPK

**Figure 5:**
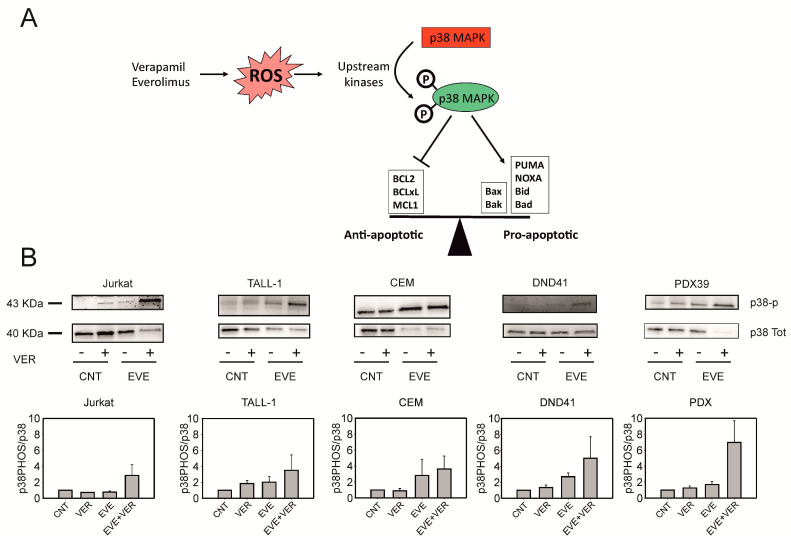
Everolimus and verapamil induce the phosphorylation of p38 MAPK. (**A**) A working model connecting ROS, p38 MAPK activation, and the induction of apoptosis induced by verapamil and everolimus. (**B**) p38 MAPK phosphorylation in T-ALL cells treated with everolimus (EVE, 10 µM) and/or verapamil (VER, 20 ng/mL) for 24 h. Shown are immunoblots from representative experiments and plots of phospho-p38/p38 ratios with means and standard error bars from three experiments.

In order to search for a mechanism to explain the potentiation of everolimus by verapamil, we investigated its effects on p38 MAPK, a member of the MAPK family that is activated in response to oxidative stress and can tip the apoptotic balance by upregulating the expression of proapoptotic “BH3-only” proteins, and by phosphorylating anti-apoptotic proteins of the BCL2 family, a modification that leads to their degradation (Figure 5A) [19]. p38 MAPK activation requires the phosphorylation of threonine 180 (T180) and tyrosine 182 (Y182) by dual-specificity kinases. Therefore, we tested the effects of everolimus and verapamil on p38 MAPK phosphorylation by immunoblotting with antibodies specific for phospho-T180 and phospho-Y182 or non-phosphorylated p38 MAPK. Results showed a substantial increase in p38 MAPK phosphorylation in response to the combination of the two drugs, while no major change was observed in cells treated with everolimus or verapamil as single agents (Figure 5B), suggesting that full engagement of this pathway requires the combined action of the two compounds.

### 3.6. Verapamil Potentiates the Effects of Everolimus In Vivo

**Figure 6:**
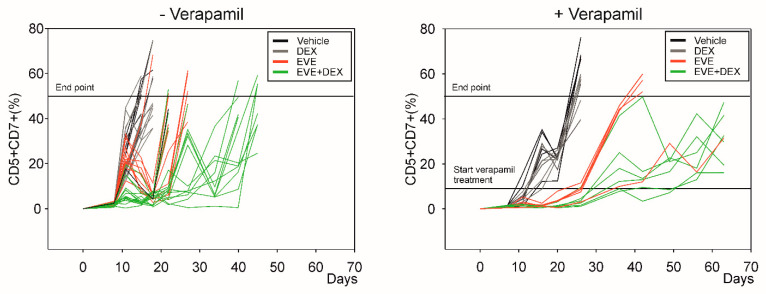
In vivo experiments with everolimus, dexamethasone, and verapamil. The 1 × 10^6^ PDX19 cells were injected in the tail vein of 24 NOD/SCID mice. After one week, the mice were treated every second day with either a drug vehicle (black lines), dexamethasone (15 mg/kg, grey lines), everolimus (4 mg/kg, red lines), or dexamethasone (15 mg/kg) plus everolimus (4 mg/kg) (green lines). The effects of drug treatments were assessed by measuring the percentages of CD5+/CD7+ PDX cells in total PBMC in the absence (**left**-hand graph) or presence (**right**-hand graph) of verapamil (20 mg/kg), which was added to the drug treatment regimen when the population of leukemic cells reached >10% (indicated by the lower horizontal line in the right-hand graph). Lines indicate individual mice.

Our previous studies indicated that everolimus increased the response of T-ALL cells to dexamethasone in vivo [7]. Therefore, we investigated whether the addition of verapamil would affect the response to everolimus plus dexamethasone. We injected 1 × 10^6^ PDX19 cells in the tail veins of NOD/SCID mice. After one week, when the percentages of the human CD5+/CD7+ blasts in the peripheral blood were 1–2%, the mice were treated with either a drug vehicle, 15 mg/kg dexamethasone, 4 mg/kg everolimus, or the drug combination. Tumor growth was assessed by measuring the percentages of CD5+/CD7+ PDX cells in total PBMC. Figure 6 shows tumor growth curves obtained in the absence (left-hand graph) or presence (right-hand graph) of verapamil, which was added to the drug treatment regimens when the population of CD5+/CD7+ leukemic cells reached >10% (indicated by the lower horizontal line in the right-hand graph). A comparison of the tumor growth curves for the individual mice and their times to reach the humane end-point (>50% of leukemia cells in the peripheral blood) indicated that the addition of verapamil substantially improved the effects of everolimus and everolimus plus dexamethasone (indicated by red and green lines, respectively).

## 4. Discussion

In the present study, we show that the calcium channel antagonist/MDR pump inhibitor verapamil synergizes with everolimus to induce cell death in T-ALL cells (Figure 2). This effect was more prominent in the T-ALL cell lines Jurkat and TALL−1, compared to CEM and DND41, and was also observed in a panel of five therapy-resistant T-ALL PDX and seven primary samples from T-ALL patients. Studies of NOD/SCID mice inoculated with refractory T-ALL PDX cells demonstrated that verapamil potentiated the anti-tumor activity of everolimus and everolimus plus dexamethasone (Figure 6). Taken together, our results provide a rationale for repurposing verapamil in association with everolimus and GC as a therapeutic avenue for refractory T-ALL.

The verapamil-everolimus combination resulted in an increase in ROS levels (Figure 4A,B and Figure S2), lipid peroxidation (Figure 4C), and protein oxidation (Figure S2). These effects are consistent with the dramatic reduction in the levels of G6PD observed in verapamil-plus-everolimus-treated cells (Figure 4D), which would lead to depletion of the pool of NADPH and thus blunt the efficacy of ROS-scavengers. Our results point toward a correlation between the cell death effects of verapamil (Figure 2) and its ability to induce changes in ROS (Figure 4), an observation that suggests that this compound might exert previously unrecognized effects on redox homeostasis. 

The everolimus-verapamil combination also induced p38 MAPK phosphorylation (Figure 5B), which, as depicted in Figure 5A, feeds into a major pro-apoptotic pathway that is induced by oxidative stress. It is interesting that while the effects of verapamil plus everolimus on p38 MAPK phosphorylation were similar among the T-ALL cell lines (Figure 5B), the effects on cell death were much more evident in Jurkat and TALL−1 cells compared to CEM and DND41 cells (Figure 2A), suggesting that the difference in the outcome of the combination treatment may depend on the status of pathway components positioned downstream from p38 MAPK phosphorylation, such as the expression levels and phosphorylation of pro- and anti-apoptotic BCL-2 family proteins (Figure 5A).

We initially hypothesized that the above-mentioned effects of verapamil in T-ALL cells were due to its well-known activity against MDR transporters of the ABC superfamily, including the MDR1/P-glycoprotein (ABCB1), ABCG2, MRP1 (ABCC1), and ABCA2 [20,21,22], which extrude chemotherapeutic drugs, including vinblastine, doxorubicin, daunorubicin, and paclitaxel, out of the cell [10]. Alterations in ABC family proteins are known to be involved in the onset of primary and secondary chemoresistance in hematological malignancies [20]. In particular, the overexpression of MRP1 (ABCC1), MRP2 (ABCC2), MRP3 (ABCC3), MRP5 (ABCC5), and MRP6 (ABCC6) correlates with a poor prognosis in acute lymphoblastic leukemia in both adults and children [21,22]. Our NGS analysis of the T-ALL cell lines Jurkat, TALL−1, and CEM revealed a common pattern of ABC family member expression, with ABCC1, a well-known verapamil target, identified as the most highly expressed ABC gene in the three cell lines (Figure 1A). The presence of MDR activity in T-ALL cells was supported by the observation that verapamil and MK 571 were similarly effective in increasing the retention of the known MDR substrate Hoechst 33342 in Jurkat cells (Figure 3C). It is worth noting that a study by Crowe and Lemaire indicated that everolimus is a substrate of P-gp in the colon cancer cell line CACO-2 [23]. However, our measurements of the effects of verapamil and MK 571 on the intracellular concentration of everolimus in the four T-ALL cell lines did not reveal a statistically significant increase in accumulation of the drug, although a trend toward increased accumulation was observed in TALL−1 cells (Figure 3A). This observation, together with the finding that verapamil was much more potent than MK 571 in killing T-ALL cells (Figure 3B), suggests that the latter effect may involve additional mechanisms, possibly unrelated to a block in the extrusion of everolimus by MDR pump activity. In cell lines that were genetically modified to over-express MRP1, verapamil was found to decrease cellular glutathione [24] by inducing the transporter to extrude glutathione, resulting in apoptotic death [25]; the property of verapamil to favor the death of cells overexpressing MDR pumps is termed ‘collateral sensitivity’ [26]. More recent studies of a highly resistant cell line overexpressing ABCB1 (P-gp/MDR1) showed that verapamil induced ATP depletion and connected the reduced ATP to a compensatory increase in ETC activity and ROS levels [27].

Could the activity of verapamil as a voltage-dependent Ca^2+^ channel (CaV) blocker influence ROS and the death of T-ALL cells? Although it is well known that altered intracellular Ca^2+^ homeostasis can act as a potent trigger of cell death [28], by blocking CaV, verapamil would be expected to blunt these effects. A recent study with patch-clamp measurements did not detect voltage-gated Ca^2+^ currents in human and mouse T-cells, but on the other hand, showed that the CaVβ1 subunit of CaV regulates T-cell function independently of channel activity [29]. Interestingly, CaV blockers, including verapamil, also inhibit several K^+^ channels, notably Kv1.3 in T-cells [30,31,32]. This might be relevant to ROS production, as inhibition of mitochondrial Kv1.3 channels is a well-documented mechanism that enhances mitochondrial ROS production [33,34]. Future studies will be aimed at investigating the influence of verapamil on Ca^2+^ and K^+^ fluxes in T-ALL cells.

## 5. Conclusions

The results of this study furnish proof-of-principle evidence for a combinatorial strategy based on verapamil, everolimus, and GC to target refractory T-ALL. All of these compounds are approved for use in humans, suggesting that the translation of this approach to the clinic might be feasible and provide a novel therapeutic option for treating refractory T-ALL, which represents a major clinical challenge at present.

## Data Availability

The RNA-seq data elaborated in this study are deposited in the NCBI’s Gene Expression Omnibus [35] and are accessible through GEO Series accession number: GSE225751 (https://www.ncbi.nlm.nih.gov/geo/query/acc.cgi?acc=GSE225751), URL accessed on 23 February 2023.

## Supplementary Materials

The following are available online at https://www.mdpi.com/article/10.3390/antiox12030625/s1, Figure S1: Verapamil enhances everolimus-induced cell death, Figure S2: Verapamil enhances oxidative stress in T-ALL cell lines, Figure S3: Quantification of G6PD in T-ALL cell lines.

## References

[1] Karrman K., Johansson B. (2017). Pediatric T-cell acute lymphoblastic leukemia. Genes Chromosomes Cancer.

[2] Bhadri V.A., Trahair T.N., Lock R.B. (2012). Glucocorticoid resistance in paediatric acute lymphoblastic leukaemia. J. Paediatr. Child. Health.

[3] Silva A., Yunes J.A., Cardoso B.A., Martins L.R., Jotta P.Y., Abecasis M., Nowill A.E., Leslie N.R., Cardoso A.A., Barata J.T. (2008). PTEN posttranslational inactivation and hyperactivation of the PI3K/Akt pathway sustain primary T cell leukemia viability. J. Clin. Investig..

[4] Silva A., Girio A., Cebola I., Santos C.I., Antunes F., Barata J.T. (2011). Intracellular reactive oxygen species are essential for PI3K/Akt/mTOR-dependent IL-7-mediated viability of T-cell acute lymphoblastic leukemia cells. Leukemia.

[5] Silic-Benussi M., Cavallari I., Vajente N., Vidali S., Chieco-Bianchi L., Di Lisa F., Saggioro D., D’Agostino D.M., Ciminale V. (2010). Redox regulation of T-cell turnover by the p13 protein of human T-cell leukemia virus type 1: Distinct effects in primary versus transformed cells. Blood.

[6] Silic-Benussi M., Cannizzaro E., Venerando A., Cavallari I., Petronilli V., La Rocca N., Marin O., Chieco-Bianchi L., Di Lisa F., D’Agostino D.M. (2009). Modulation of mitochondrial K(+) permeability and reactive oxygen species production by the p13 protein of human T-cell leukemia virus type 1. Biochim. Biophys. Acta.

[7] Silic-Benussi M., Sharova E., Ciccarese F., Cavallari I., Raimondi V., Urso L., Corradin A., Kotler H., Scattolin G., Buldini B. (2022). mTOR inhibition downregulates glucose-6-phosphate dehydrogenase and induces ROS-dependent death in T-cell acute lymphoblastic leukemia cells. Redox Biol..

[8] Evangelisti C., Chiarini F., McCubrey J.A., Martelli A.M. (2018). Therapeutic Targeting of mTOR in T-Cell Acute Lymphoblastic Leukemia: An Update. Int. J. Mol. Sci..

[9] Gottesman M.M., Pastan I.H. (2015). The Role of Multidrug Resistance Efflux Pumps in Cancer: Revisiting a JNCI Publication Exploring Expression of the MDR1 (P-glycoprotein) Gene. J. Natl. Cancer Inst..

[10] Tsuruo T., Iida H., Tsukagoshi S., Sakurai Y. (1981). Overcoming of vincristine resistance in P388 leukemia in vivo and in vitro through enhanced cytotoxicity of vincristine and vinblastine by verapamil. Cancer Res..

[11] Agnusdei V., Minuzzo S., Frasson C., Grassi A., Axelrod F., Satyal S., Gurney A., Hoey T., Seganfreddo E., Basso G. (2014). Therapeutic antibody targeting of Notch1 in T-acute lymphoblastic leukemia xenografts. Leukemia.

[12] Silic-Benussi M., Scattolin G., Cavallari I., Minuzzo S., Del Bianco P., Francescato S., Basso G., Indraccolo S., D’Agostino D.M., Ciminale V. (2018). Selective killing of human T-ALL cells: An integrated approach targeting redox homeostasis and the OMA1/OPA1 axis. Cell Death Dis..

[13] Love M.I., Huber W., Anders S. (2014). Moderated estimation of fold change and dispersion for RNA-seq data with DESeq2. Genome Biol..

[14] Benjamini Y., Hochberg Y. (1995). Controlling the False Discovery Rate: A Practical and Powerful Approach to Multiple Testing. J. R. Stat. Society. Ser. B Methodol..

[15] Vasiliou V., Vasiliou K., Nebert D.W. (2009). Human ATP-binding cassette (ABC) transporter family. Hum. Genom..

[16] Di Veroli G.Y., Fornari C., Wang D., Mollard S., Bramhall J.L., Richards F.M., Jodrell D.I. (2016). Combenefit: An interactive platform for the analysis and visualization of drug combinations. Bioinformatics.

[17] Chou T.C. (2010). Drug combination studies and their synergy quantification using the Chou-Talalay method. Cancer Res..

[18] Telford W.G., Bradford J., Godfrey W., Robey R.W., Bates S.E. (2007). Side population analysis using a violet-excited cell-permeable DNA binding dye. Stem. Cells.

[19] Whitaker R.H., Cook J.G. (2021). Stress Relief Techniques: p38 MAPK Determines the Balance of Cell Cycle and Apoptosis Pathways. Biomolecules.

[20] Hofmann W.K., Trumpp A., Müller-Tidow C. (2023). Therapy resistance mechanisms in hematological malignancies. Int. J. Cancer.

[21] Plasschaert S.L., de Bont E.S., Boezen M., vander Kolk D.M., Daenen S.M., Faber K.N., Kamps W.A., de Vries E.G., Vellenga E. (2005). Expression of multidrug resistance-associated proteins predicts prognosis in childhood and adult acute lymphoblastic leukemia. Clin. Cancer Res..

[22] Steinbach D., Wittig S., Cario G., Viehmann S., Mueller A., Gruhn B., Haefer R., Zintl F., Sauerbrey A. (2003). The multidrug resistance-associated protein 3 (MRP3) is associated with a poor outcome in childhood ALL and may account for the worse prognosis in male patients and T-cell immunophenotype. Blood.

[23] Crowe A., Lemaire M. (1998). In vitro and in situ absorption of SDZ-RAD using a human intestinal cell line (Caco-2) and a single pass perfusion model in rats: Comparison with rapamycin. Pharm. Res..

[24] Cullen K.V., Davey R.A., Davey M.W. (2001). Verapamil-stimulated glutathione transport by the multidrug resistance-associated protein (MRP1) in leukaemia cells. Biochem. Pharmacol..

[25] Trompier D., Chang X.B., Barattin R., du Moulinet D’Hardemare A., Di Pietro A., Baubichon-Cortay H. (2004). Verapamil and its derivative trigger apoptosis through glutathione extrusion by multidrug resistance protein MRP1. Cancer Res..

[26] Pluchino K.M., Hall M.D., Goldsborough A.S., Callaghan R., Gottesman M.M. (2012). Collateral sensitivity as a strategy against cancer multidrug resistance. Drug Resist. Updat..

[27] Gao X., Aguanno D., Board M., Callaghan R. (2021). Exploiting the metabolic energy demands of drug efflux pumps provides a strategy to overcome multidrug resistance in cancer. Biochim. Biophys. Acta Gen. Subj..

[28] Pinton P., Giorgi C., Siviero R., Zecchini E., Rizzuto R. (2008). Calcium and apoptosis: ER-mitochondria Ca^2+^ transfer in the control of apoptosis. Oncogene.

[29] Erdogmus S., Concepcion A.R., Yamashita M., Sidhu I., Tao A.Y., Li W., Rocha P., Huang B., Garippa R., Lee B. (2022). Cavβ1 regulates T cell expansion and apoptosis independently of voltage-gated Ca^2+^ channel function. Nat. Commun..

[30] Pancrazio J.J., Viglione M.P., Kleiman R.J., Kim Y.I. (1991). Verapamil-induced blockade of voltage-activated K+ current in small-cell lung cancer cells. J. Pharmacol. Exp. Ther..

[31] Chandy K.G., Wulff H., Beeton C., Pennington M., Gutman G.A., Cahalan M.D. (2004). K+ channels as targets for specific immunomodulation. Trends Pharmacol. Sci..

[32] Cahalan M.D., Chandy K.G. (2009). The functional network of ion channels in T lymphocytes. Immunol. Rev..

[33] Leanza L., Henry B., Sassi N., Zoratti M., Chandy K.G., Gulbins E., Szabò I. (2012). Inhibitors of mitochondrial Kv1.3 channels induce Bax/Bak-independent death of cancer cells. EMBO Mol. Med..

[34] Szabo I., Zoratti M., Biasutto L. (2021). Targeting mitochondrial ion channels for cancer therapy. Redox Biol..

[35] Edgar R., Domrachev M., Lash A.E. (2002). Gene Expression Omnibus: NCBI gene expression and hybridization array data repository. Nucleic Acids Res..

