# Supplementary material for: Repurposing Verapamil to Enhance Killing of T-ALL Cells by the mTOR Inhibitor Everolimus
Source: Antioxidants (Basel). 2023 Mar 3;12(3):625. doi: 10.3390/antiox12030625 (PMC10045900; doi:10.3390/antiox12030625)
Supplement: Supplementary file 1 [file antioxidants-12-00625-s001.zip › antioxidants-2201795-supplementary.pdf]

Supplementary material

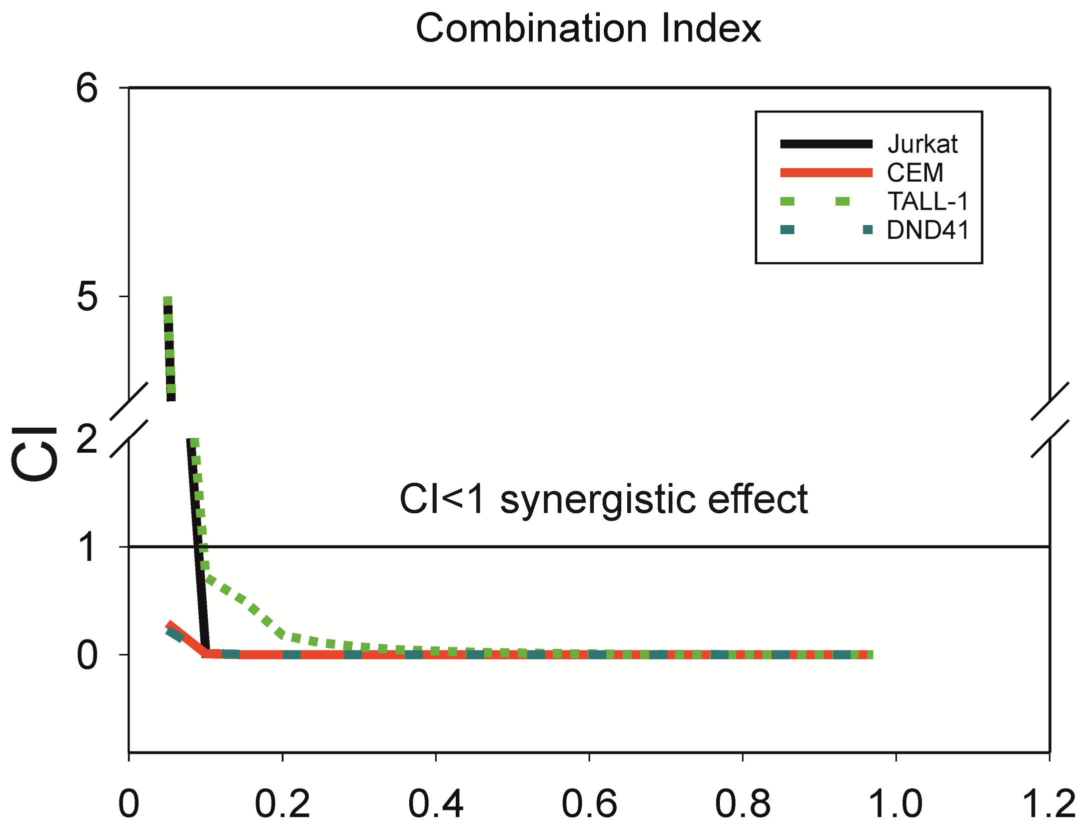

Figure S1. Verapamil enhances everolimus-induced cell death

The combination index of everolimus and verapamil in Jurkat, CEM, TALL-1 and DND1 (black, red, green and cyan lines, respectively) was calculated using CompuSyn software. The FA (fraction affected) was calculated using specific cell death values measured as percentages of PI-positive cells.

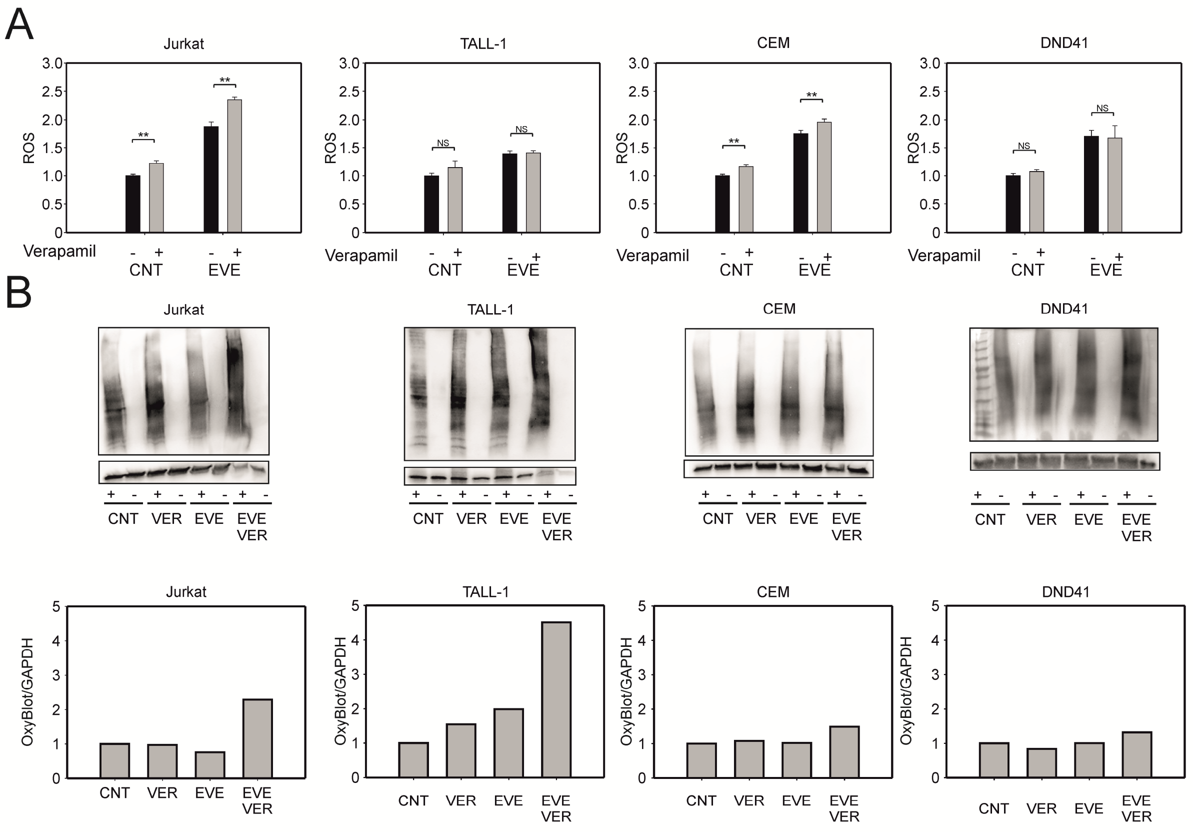

Figure S2. Verapamil enhances oxidative stress in T-ALL cell lines

(A) Detection of intracellular ROS using the ROS-Glo H_2O_2 bioluminescent assay (Promega). Jurkat, TALL-1, CEM and DND1 cells were treated for 24 hrs with drugs as indicated in the figure. After incubation 5x105 cells were collected, washed in PBS, resuspended in H2O2 substrate diluent buffer (20 µl H2O2 substrate buffer, 0.25 µl of H2O2 substrate) and incubated at 37°C for 4 hrs in a 96-well white plate. ROS-Glo detection solution (100 µL luciferin detection reagent, 1 µL D-cysteine, 1 µL signal enhancer solution) was added to each well and after 20 minutes' incubation at room temperature the plate was read with a Victor NIVO plate reader (PerkinElmer). Graphs show resulting signals after normalization against the signal obtained for vehicle-treated cells. (B) Detection of protein oxidation with the Oxyblot kit (Merck). The Oxyblot method is based on the detection of carbonyl groups introduced into proteins by oxidative reactions. Cells were treated with drugs for 24 hrs as indicated in the figure, washed with PBS, and lysed in cell disruption buffer containing 50 mM dithiothreitol

(DTT). Equal aliquots of lysates (15-30 µg) were treated with 2,4-dinitrophenylhydrazine (DNPH) to add dinitrophenylhydra-zone (DNP-hydrazone) groups to oxidized amino acids or with the kit's negative control solution according to the OxyBlot protocol. Samples were subjected to SDS-PAGE in 4-20% Mini-PROTEAN TGX gels and then transferred to nitrocellulose membranes (GE Healthcare). Membranes were saturated overnight with 5% BSA in TBS-Tween 20 and incubated with rabbit anti-DNP (1:150) and HRP-conjugated anti-rabbit antibody (1:300) provided in the OxyBlot kit. Chemiluminescent signals were detected using Lite Ablot Turbo or PURECL Dura (Vilber Lourmat) and a Cambridge UVITEC imaging system. Blots were then processed to detect GAPDH as described in the Material and Methods section. (+) and (–) symbols below images indicate DNPH-treated and control-treated samples, respectively. The bar graphs show quantification of oxidized protein bands normalized against the GAPDH signal in each lane.

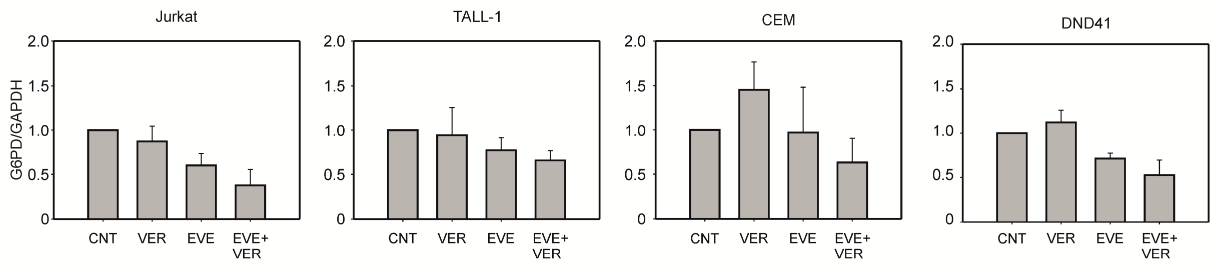

Figure S3. Quantification of G6PD in T-ALL cell lines

T-ALL cell lines were treated and analyzed by immunoblotting to detect G6PD and GAPDH as described in the Material and Methods section and in the legend to Figure 4D. The graphs show the quantification of the mean G6PD/GAPDH ratios and standard error bars from 3 independent experiments.
